# Nisin-Loaded Ulvan Particles: Preparation and Characterization

**DOI:** 10.3390/foods10051007

**Published:** 2021-05-04

**Authors:** Ruta Gruskiene, Tatjana Kavleiskaja, Ramune Staneviciene, Stefanos Kikionis, Efstathia Ioannou, Elena Serviene, Vassilios Roussis, Jolanta Sereikaite

**Affiliations:** 1 Department of Chemistry and Bioengineering, Vilnius Gediminas Technical University, 10221 Vilnius, Lithuania; ruta.gruskiene@vgtu.lt (R.G.); serviene@gmail.com (E.S.); 2 Institute of Chemistry, Vilnius University, 01513 Vilnius, Lithuania; tania.krivorot81@gmail.com; 3 Laboratory of Genetics, Institute of Botany, Nature Research Centre, 08412 Vilnius, Lithuania; ramune.staneviciene@gamtc.lt; 4 Section of Pharmacognosy and Chemistry of Natural Products, Department of Pharmacy, National and Kapodistrian University of Athens, Panepistimiopolis Zografou, 15771 Athens, Greece; skikionis@pharm.uoa.gr (S.K.); eioannou@pharm.uoa.gr (E.I.); roussis@pharm.uoa.gr (V.R.)

**Keywords:** nisin, ulvan, encapsulation, antimicrobial activity

## Abstract

Nisin is an attractive alternative to chemical preservatives in the food industry. It is a cationic peptide of 34 amino acid residues that exhibits antimicrobial activity against Gram-positive bacteria. To ensure nisin stability in food matrices, new nisin-loaded ulvan particles were developed by the complexation method. The interaction of nisin with ulvan was demonstrated by FT-IR spectroscopy and differential scanning calorimetry. The encapsulation efficiency was calculated at different pH values within the range of 4.0–7.0 and was found to have the highest value at pH 7.0. The size and surface charge of particles fabricated at different concentrations of nisin and pH values were determined. Nisin-loaded ulvan particles exhibited antimicrobial activity against Gram-positive bacteria comparable to that of free nisin. Therefore, the developed complexes have the potential for application as biopreservatives in the food industry. For the first time, the potential of ulvan as a carrier of antimicrobial agent nisin was demonstrated.

## 1. Introduction

Foodborne diseases have a major socio-economic impact, and special attention has been paid to the development of various technologies for the microbial control of food, such as high-pressure processing, pulsed electric field processing, food irradiation and plasma processing. Nevertheless, thermal processing and the preservation of food using chemicals remain the most widely applied methods [1]. However, the demand of consumers for safe and healthy food is constantly increasing, leading to the development of novel preservation methods, such as biopreservation [2]. 

Bacteriocins are natural antimicrobial agents and attractive alternatives to chemical preservatives. They are proteins or peptides produced by Gram-positive and Gram-negative bacteria [3,4]. Nisin produced by *Lactococcus lactis* subsp. *lactis* is the most popular preservative in the food industry. It is a cationic peptide composed of 34 amino acid residues, belonging to the class I bacteriocins named lantibiotics. Nisin is approved for use by the US Food and Drug Administration and is also recognized as a food additive in the EU (assigned as E234). Nisin is highly active against Gram-positive bacteria, including *Listeria monocytogenes* and *Clostridium botulinum* which are known to be pathogenic [5]. Gram-positive bacteria exhibit a natural variation in their sensitivity towards nisin [6,7], which forms a complex with Lipid II molecule as a receptor, following pore formation and membrane permeabilization [8]. The pore complex consists of four lipid II and eight nisin molecules. Lipid II is an essential cell wall precursor, and nisin binding blocks the synthesis of the cell wall [9,10,11]. However, nisin is ineffective against Gram-negative bacteria due to the impermeability of their outer membrane [12]. Gram-negative bacteria may be inactivated by the use of nisin in combination with physical methods, such as high hydrostatic pressure or pulsed electric fields, which induce the permeabilization of their outer membrane [13,14]. 

The merits of bacteriocins can be limited due to their interaction with the food matrix resulting in an uneven distribution in the food products. Furthermore, bacteriocins as peptides or proteins can easily lose their activity due to proteolytic degradation. If pure bacteriocins are directly added to food, their long-term stability is debatable [15]. To overcome such shortcomings, there is a great demand for the development of new and effective delivery systems, and the application of encapsulation technology has seriously been considered. Biopolymeric materials based on proteins and polysaccharides are frequently used for the preparation of nano/microparticles loaded with bioactive agents. Biopolymers from natural sources have inherent low toxicity and high stability and are suitable for application in the food industry [16,17,18]. 

Ulvan is a water-soluble sulfated polysaccharide isolated from green algae belonging to the genus *Ulva,* which are edible seaweeds with a wide range of health-promoting properties. Moreover, they consist of renewable biomass and can be used as an inexpensive source of polysaccharides regarded as safe for application in the human diet. The carbohydrate composition of ulvan is complex and variable and depends on the particular green algal species, growth conditions and harvesting region and period, as well as the extraction and purification methods. Ulvan is a cell wall polysaccharide with a backbone composed of α- and β-(1,4)-linked monosaccharides, namely rhamnose, xylose, glucuronic acid and iduronic acid. The sulfation sites are localized mainly on rhamnose [19,20,21]. Ulvan exhibits many biological activities, including antiviral, antioxidant, anticancer, antibacterial, antihyperlipidemic, anticoagulant and immunostimulating activities [22,23]. Ulvan, due to its valuable biological properties, has the potential to be evolved as a beneficial ingredient in food, pharmaceutical and nutraceutical products.

Herein, we describe a simple and cost-efficient way for encapsulation of nisin using ulvan. New nisin-loaded ulvan particles can serve as antimicrobials that can also possess the above-mentioned biological properties of ulvan. 

## 2. Materials and Methods

### 2.1. Materials

Nisin (NisinZ P) was obtained from Handary S.A. (Brussels, Belgium). Hydrochloric acid (32%, for analytical purposes, p.a.), sodium hydroxide (≥98%, p.a.) and ortho-phosphoric acid (85%, p.a.) were purchased from Carl Roth (Karlsruhe, Germany). Polyvinyl alcohol (average MW 22000) was obtained from Fluka (Buchs, Switzerland). Specimens of the green alga *Ulva rigida* were collected at the island of Evoia, Greece. The specimens were cleaned from epiphytes, washed with seawater and fresh water, air-dried, milled in 1–5 mm pieces and stored, until used, at room temperature in paper bags in a dark, dry place. For the extraction of ulvan, an aquatic suspension of 500 g of the air-dried alga in 10 L of distilled water was heated in an autoclave for 30 min at 121 °C and subsequently filtered through cotton cloth to afford a hot aqueous solution that was allowed to cool at room temperature. The polysaccharide was subsequently precipitated by the addition of 40 L ethanol (96% *v*/*v*), and the resulting suspension was left overnight at 4 °C. The precipitate was filtered through cotton cloth, washed with ethanol, sonicated in an ultrasonic bath, vacuum-filtered and finally freeze-dried to afford ulvan as an off-white powder. The chemical analyses of ulvan were performed as previously described [24]. The analysis of ulvan, with a molecular weight distribution centered at 1670 kDa, revealed 39.6% carbohydrates, 48.8% sulfate, 0.5% protein and 11.1% ash on a dry weight basis. All reagents were used as obtained without additional purification.

### 2.2. Preparation of Nisin-Loaded Ulvan Particles

The stock solutions of both ulvan and nisin were prepared in deionized water at the concentration of 1 mg/mL and filtered through 0.2 μm pore size filters. Then, a pH value in the range of 4.0–7.0 was adjusted using 0.1 M NaOH or 0.1 M HCl. To form particles, the solution of nisin was added dropwise to the ulvan solution under constant stirring at room temperature. Before use, the solution of ulvan was diluted with deionized water to obtain 0.4 mg/mL of ulvan in the final nisin–ulvan mixture of 3 mL. The final concentration of nisin was in the range of 0.0–1.0 mg/mL. Finally, the pH value of the mixture was additionally adjusted to 4, 5, 6 and 7. 

### 2.3. Determination of Nisin Encapsulation Efficiency

A volume of 0.25 mL of particle solution was centrifuged at 10,000× *g* at 4 °C for 15 min using ultra-filtration tubes (ROTI Spin MINI-10, cut-off 10 kDa). The concentration of free nisin in the solution of the outer tube was measured by capillary zone electrophoresis (CZE) method using 7100 Capillary Electrophoresis unit (Agilent Technologies). CZE was run at 20 °C applying bare fused silica capillary (50 mm ID, 64.5 cm total length, 56 cm effective length). Run buffer was 50 mM phosphate pH 2.5 containing 0.05% polyvinyl alcohol. For the sample introduction into the capillary, the hydrodynamic injection at 50 mbar for 4 s was applied. Separation was carried out at 30 kV with positive polarity at the inlet. The detection of nisin was performed at 200 nm. For the calculation of the percentage of residual free nisin, peak areas of the solution in the outer tube and blank sample containing all components except ulvan were compared (Figures S1 and S2). The efficiency of nisin encapsulation (EE) was calculated using the following equation:

EE (%) = ((Total nisin − free nisin)/Total nisin) × 100

### 2.4. FT-IR Spectroscopic Analysis

Fourier transform infrared (FT-IR) spectra were recorded using an FT-IR spectrophotometer (PerkinElmer Frontier FT-IR) under the following conditions: a scan range of 550–4000 cm^−1^, a resolution of 4 cm^−1^ and 500 scans. 

### 2.5. Physicochemical Characterization of Nisin-Loaded Ulvan Particles

The Zetasizer NanoZS (Malvern Instruments) instrument equipped with a 4 mV HeNe laser at a wavelength of 633 nm was used for the determination of the hydrodynamic diameter of particles. The intensity of the scattered light was measured at the angle of 173° and 20 °C. The size distributions were obtained from the correlation functions. The Malvern Zetasizer software 7.03 was used for the analysis of data. 

The zeta potential of particles was determined using the instrument mentioned above. The Smoluchowski equation was used to convert the electrophoretic mobility to zeta potential.

Thermal properties of complexes were determined using differential scanning calorimetry and thermogravimetric analysis as previously described [25]. Briefly, differential scanning calorimetry (DSC) analysis was carried out using a TA Thermal Analyzer (Discovery DSC 25, TA Instruments, New Castle, DE, USA). Sealed samples of 5–7 mg in aluminum pans were heated at a constant rate of 10 °C/min from 40 to 350 °C under a 25 mL/min flow of nitrogen. Thermogravimetric analysis (TGA) was performed using a TA Thermogravimetric Analyzer (TGA 55, TA Instruments, New Castle, DE, USA) under nitrogen flow of 25 mL/min at a heating rate of 10 °C/min from 40 to 600 °C. 

A Phenom-World desktop scanning electron microscope (SEM, Thermo Fischer Scientific, Waltham, MA, USA) with a tungsten filament (10 kV) and charge reduction sample holder was used for SEM analyses of the freeze-dried nisin-loaded ulvan particles without sputter coating.

### 2.6. Bacteria Cultures and Growth Conditions

Gram-positive bacteria *Bacillus subtilis* ATCC 6633 (Vilnius University, Lithuania) and *Listeria innocua* CECT 910T (kindly provided by Maria Joao Fraqueza, University of Lisbon, Portugal) and Gram-negative bacteria *Escherichia coli* BL21 (F-dcm ompT hsdS (rB-mB-) gall (DE3)) (Thermo Fisher Scientific, Lithuania) and *Klebsiella pneumoniae* KV-3 (Vilnius University, Lithuania) were used in this study. Bacteria were propagated in Luria–Bertani (LB) medium (2% tryptone, 2% yeast extract, 1% NaCl) for 16–18 h under continuous shaking at 37 °C.

### 2.7. Analysis of Antimicrobial Activity by Agar Diffusion Assay

The antimicrobial activity and minimum inhibitory concentration (MIC) of nisin-loaded particles were tested using the agar diffusion assay against Gram-positive and Gram-negative bacteria. For the measurement of the antimicrobial activity, 10 µL of particles obtained at the nisin concentration of 0.2 mg/mL and free nisin samples were spotted on the LB agar, pH 6.0, plates seeded with overnight grown *B. subtilis, E. coli, K. pneumoniae* or *L. innocua* cultures (2 × 10^7^ cells/plate). The plates were incubated at 37 °C for 24 h, and growth inhibition zones were measured.

For the determination of MIC, nisin-loaded particles and free nisin solution were serially 2-fold diluted in sterile H_2_O. Volumes of 10 µL of each tested substance with different concentrations of nisin were spotted on the LB agar, pH 6.0, seeded with stationary bacteria cells (2 × 10^7^ cells/plate) of interest and incubated at 37 °C for 24 h. MIC was determined as the minimal concentration of nisin with a visible inhibition zone.

### 2.8. Analysis of the Efficiency of Nisin-Loaded Ulvan Particles in Carrot Juice

Carrots were purchased from a local food store (Vilnius, Lithuania) and transferred into the laboratory. Then, they were washed with tap water, peeled and grated. The fresh juice was squeezed, sterilized for 10 min at 80 °C and kept in a sterile Falcon tube at 4 °C until further use. Overnight grown *B. subtilis* and *L. innocua* cells were collected by centrifugation at 6000× *g* for 5 min, washed 2 times with 0.9% NaCl solution and resuspended in 0.9% NaCl at the final concentration of about 1 × 10^9^ cells/mL. The cells (150 µL) were mixed with 150 µL of carrot juice or 0.9% NaCl solution, and 60 µL of nisin-loaded particles solution prepared at the nisin and ulvan concentrations of 0.2 and 0.4 mg/mL, respectively, at pH 6.0 was added. Samples were incubated at room temperature (20 °C) for 24 h. Serial dilutions were performed in 0.9% NaCl, and 50 µL of each solution was spread onto LB agar plates, which was followed by overnight incubation at 37 °C and counting of colony-forming units (CFUs). All experiments were performed in triplicate, and the mean value of CFU/mL was calculated. 

### 2.9. Statistical Analysis

One-way analysis of variance (ANOVA, *p* < 0.05) was used to define statistically significant results. The data are presented as means ± standard deviations of three independent experiments.

## 3. Results and Discussion

### 3.1. Preparation and Physicochemical Characterization of Nisin-Loaded Ulvan Particles 

**Figure 1:**
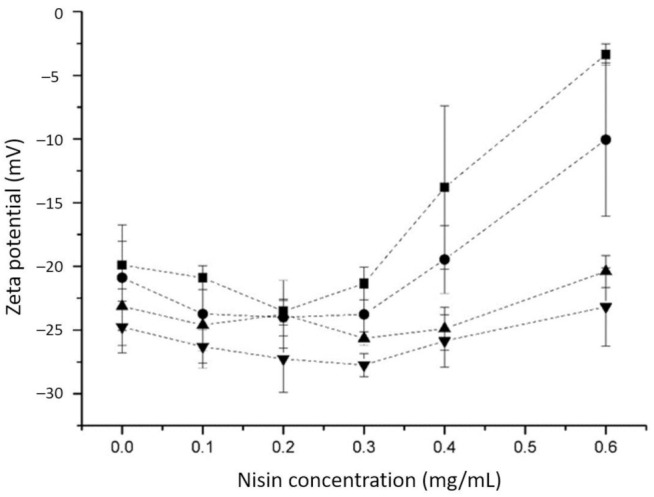
Zeta potential of nisin-loaded ulvan particles at different pH values depending on nisin concentration; -■-, pH 4.0; -*●*-, pH 5.0; -▲-, pH 6.0; -▼-, pH 7.0. The final concentration of ulvan was equal to 0.4 mg/mL. Data are shown as mean values ± standard deviations of three parallel measurements.

In this work, nisin-loaded ulvan particles were prepared by the complexation method. The process was performed at different pH values in the range of 4.0–7.0. The zeta potential of ulvan slightly decreased (increased in the absolute value) with increasing pH value from 4 to 7 (Figure 1). This is related to the deprotonation of uronic acids (pK_a_ ~3.28), which are present in the structure of ulvan [20]. Nisin is a cationic peptide with an isoelectric point above 8.5 [26]. The addition of nisin caused the reduction in the negative surface charge and the complex formation. This tendency was most observable at pH values of 4 and 5. However, at lower nisin concentration, the addition of nisin caused a slight increase in the negative surface charge of particles, which could be related to the conformational changes of ulvan due to the interaction with nisin molecules. The formation of the complex by electrostatic interaction depends not only on the net opposite charge and the size of macromolecules but also on their flexibility [27,28] 

**Figure 2:**
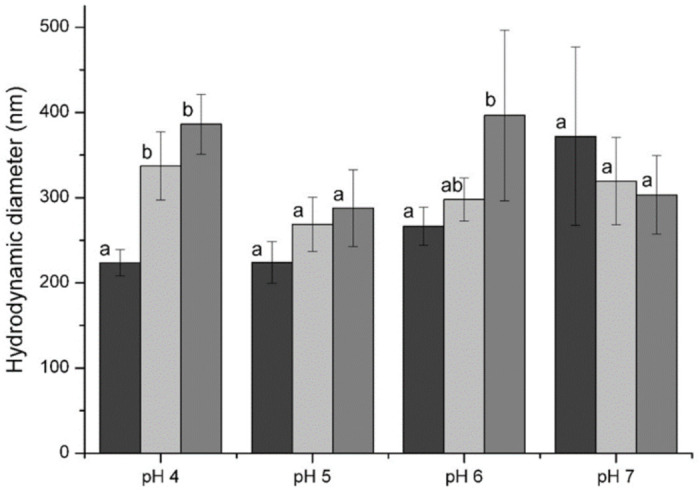
Size of nisin-loaded ulvan particles at different pH values depending on nisin concentration: 0.0 mg/mL—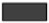; 0.1 mg/mL—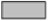; 0.2 mg/mL—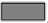. Final concentration of ulvan in the solution was equal to 0.4 mg/mL. Different letters indicate significant differences (*p* < 0.05) in the mean of the size of particles within the group of each pH value.

**Figure 3:**
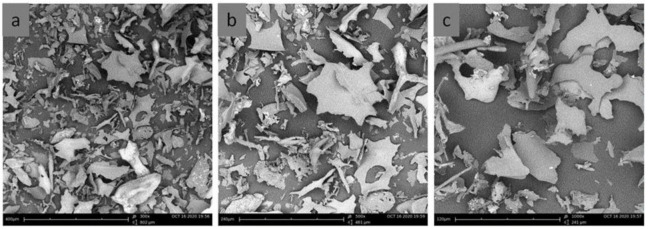
SEM images of the freeze-dried nisin-loaded ulvan particles at (**a**) ×300, (**b**) ×500 and (**c**) ×1000 magnification. For particle preparation, the concentrations of ulvan and nisin were 0.4 mg/mL at pH 6.0.

The addition of nisin at low concentrations did not induce large changes in the hydrodynamic diameter of particles (Figure 2) except at pH 4. As mentioned above, the increase in negative surface charge and possible conformation rearrangements could be the reason for the size increase. However, at the nisin concentrations above 0.2 mg/mL, the particles became larger and precipitated from the solution at pH 4.0. At the nisin concentration above 0.3 mg/mL, the particles precipitated at all pH values used in the experiment. SEM image analysis revealed the morphological characteristics of the freeze-dried nisin-loaded ulvan particles. The formed inclusion particles were randomly distributed in complexes of various sizes exhibiting irregular shapes of either a rock-like or flake-like morphology, indicating the formation of agglomerates (Figure 3).

The nisin encapsulation efficiency was determined by the method of CZE. As seen from Table 1, it was dependent on pH value and found to be 100% up to nisin concentration of 0.3 mg/mL at all pH values in the range of 4.0–7.0. The subsequent increase in the nisin concentration, especially at lower pH values, was followed by the incomplete encapsulation of the bacteriocin. The lowest nisin encapsulation efficiency was found at pH 4.0, and the highest one was at pH 7.0. Despite the reduction in nisin positive charge with increasing pH value, the encapsulation efficiency was about 92% in the presence of 1.0 mg/mL nisin at pH 7.0 (Table 1). Previously, it was demonstrated that nisin interaction with polyanionic polysaccharides depends on not only electrostatic but also hydrophobic forces [29]. Nisin is an amphiphilic peptide. The N-terminal part of the peptide chain has a relatively high number of hydrophobic residues [30]. On the other hand, ulvan has a high number of methyl groups in the repeating units of rhamnose [19]. Therefore, the contribution of hydrophobic interactions to the complex formation is significant. 

**Figure 4:**
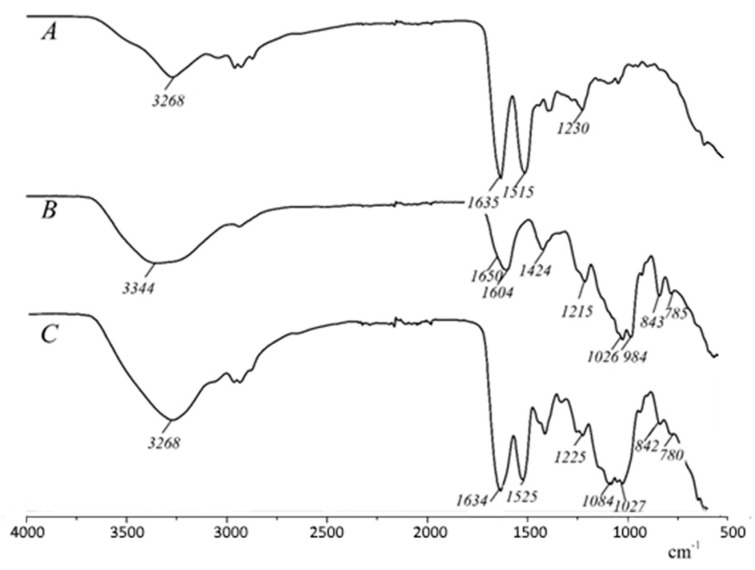
FT-IR spectra of nisin (**A**), ulvan (**B**) and ulvan–nisin complex prepared at pH 6.0 (**C**).

FT-IR spectroscopy was used to investigate the interaction between nisin and ulvan. Figure 4 represents the FT-IR spectra of nisin, ulvan and their complex. In the nisin spectrum, a characteristic absorption peak centered at approximately 3268 cm^−1^ is assigned to the stretching vibration of hydrogen-bonded –OH and NH. Three characteristic absorption peaks at approximately 1635, 1515 and 1230 cm^−1^ correspond to amide I, amide, II and amide III bands, respectively, and are assigned to the secondary structure of peptides [31,32]. For ulvan, the broad band at 3500−3200 cm^−1^ corresponds to stretching of O–H groups. The carboxyl groups in the acidic moieties show a symmetric peak with the stretching maximum at 1604 cm^−1^ and asymmetric stretching at 1424 cm^−1^. The symmetric peak at 1604 cm^−1^ has a shoulder at 1650 cm^−1^, suggesting the presence of S=O stretching of sulfate groups. Two other peaks at 843 and 785 cm^−1^ also belong to sulfate groups and are characteristic of sulfated polysaccharides [33,34]. The broad peak around 1020−1050 cm^−1^ corresponds to symmetric stretching of C–O–C linkages. The increase in the intensity and the shift of the broad peak of O–H stretching from 3344 to 3268 cm^−1^ indicate the involvement of O–H groups in the formation of the nisin–ulvan complex. The more intense peak of amide I overlaps with the peaks of the carboxyl and sulfate groups of ulvan in the spectrum of the complex. The shift of the amide I peak is not observed, which indicates the absence of configuration changes interacting with the anionic polysaccharide [35]. Meanwhile, the peak of amide II (NH bending and CN stretching) shifted from 1515 cm^−1^ to the higher wavenumber of 1525 cm^−1^ during the formation of new hydrogen bonds [36]. After the complexation, the peaks in the ulvan spectrum at 1026 and 984 cm^−1^ shifted to 1084 and 1027 cm^−1^, respectively. This region of carbohydrates corresponding to stretching of C–O–C linkages is influenced by the change of steric arrangements and interactions of axial and equatorial C–OH groups [37]. 

**Figure 5:**
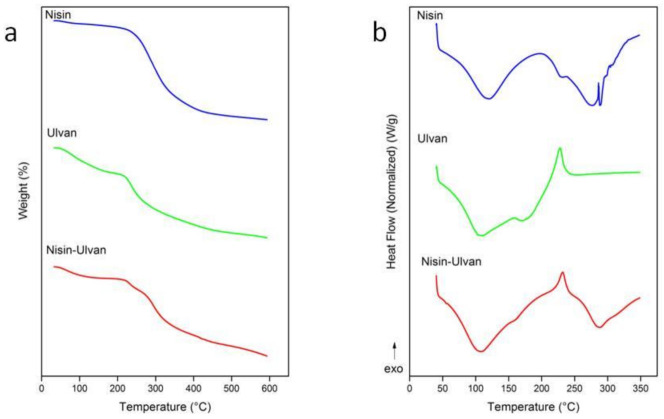
TGA (**a**) and DSC (**b**) thermograms of nisin, ulvan and nisin-loaded ulvan particles at pH 6.0.

Differential scanning calorimetry (DSC) and thermogravimetric analysis (TGA) were used to investigate the thermal properties of nisin, ulvan and nisin-loaded ulvan particles. In the TGA, nisin exhibited one main degradation step with the major degradation peak at 286 °C. Ulvan exhibited an initial dehydration mass loss and started to degrade at 224 °C. The composite of the two materials revealed two main degradation shifts, one at 224 °C and the second at 286 °C. (Figure 5a). In the DSC analysis, nisin showed an endotherm at 129.8 °C correlated to the loss of moisture, followed by two major exothermic peaks, namely one broad exothermic peak at 202.2 °C as a result of degradation and depolymerization events and one sharp exothermic peak at 286 °C associated with decomposition phenomena. The thermogram of ulvan showed the characteristic thermal behavior of polysaccharides with a broad endotherm at 105.4 °C followed by a higher decomposition exothermic peak at 227.7 °C. In the case of nisin–ulvan composite, a broad endotherm was observed at 106.5 °C, followed by a broad exothermic peak at 231.7 °C attributed to the degradation of the composite. The thermogram pattern of the composite is related to the patterns of the thermograms of the two combined ingredients. The shifted degradation exotherm of ulvan from 227.7 to 231.7 °C and the absence of the characteristic exothermic peak of nisin at 286 °C suggest the molecular interaction of the two components, forming a new material with a difference in structure and functional groups and, therefore, different thermal behavior (Figure 5b).

### 3.2. Antimicrobial Activity of Nisin-Loaded Ulvan Particles

**Figure 6:**
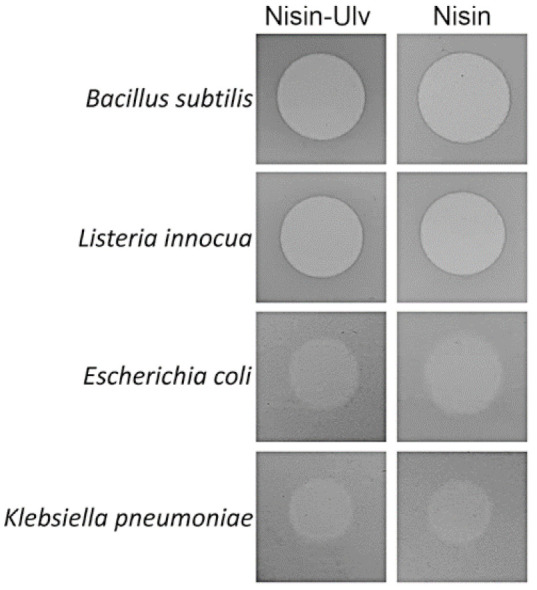
Bacteria sensitivity to nisin-loaded nanoparticles. The inhibitory activity of encapsulated (Nisin-Ulv) and free nisin was demonstrated by measuring growth inhibition zones in agar diffusion assay. Free nisin concentration was 0.2 mg/mL; nisin-loaded ulvan particles were prepared using the same concentration of nisin.

**Figure 7:**
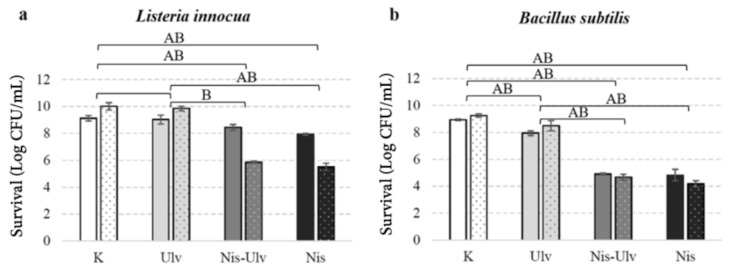
The viability of *L. innocua* (**a**) and *B. subtilis* (**b**) under the action of nisin-loaded ulvan (Nis-Ulv) particles, free ulvan (Ulv) or nisin solution (Nis) performed in carrot juice (columns with dots) and 0.9% NaCl solution (columns without dots). In control experiments (K), bacterial cells in carrot juice or 0.9% NaCl were incubated without additives. The data are given as log CFU/mL survival. Significant differences (*p* < 0.05) between the survival of the indicator organisms in the control and the treatment groups are marked A and B in 0.9% NaCl solution and juice, respectively. The absence of a letter means insignificant differences.

The antibacterial activity of prepared particles against the Gram-positive (*Bacillus subtilis* and *Listeria innocua*) and Gram-negative (*Escherichia coli* and *Klebsiella pneumoniae*) bacterial strains was assessed by agar diffusion and/or survival assays (Figure 6 and Figure 7). The minimum inhibitory concentration (MIC) was determined for tested free and encapsulated nisin; the values (µg/mL) are indicated in Table 2. The evaluation of the antibacterial activity showed that the antibacterial activity of encapsulated nisin was not reduced as compared to that of free nisin. Nisin-loaded ulvan particles exhibited the highest antibacterial activity against the Gram-positive bacterium *B. subtilis.* According to the agar diffusion assay, the largest inhibitory zone of 11–12 mm in diameter was observed, and MIC was determined at 0.024 µg/mL. Based on the viability assay, the application of nisin–ulvan particles resulted in about 4 log CFU/mL reduction in *B. subtilis* cells (Figure 7). The antibacterial effect of encapsulated and free nisin against *L. innocua* was weaker in comparison to *B. subtilis* (Figure 6 and Figure 7). The MIC values increased about 65 times and reached 0.781 and 1.563 µg/mL for nisin and nisin–ulvan samples, respectively (Table 2). The CFU reduction reached about 1 log using nisin-loaded ulvan particles or free nisin (Figure 7). This is in good agreement with previously published data demonstrating the higher sensitivity of *B. subtilis* to nisin as compared with *L. innocua* [38]. On the contrary, free nisin was slightly more effective against Gram-positive bacteria in comparison to nisin-loaded particles. The lower antimicrobial activity could be related to the slow release process of encapsulated nisin. 

The antibacterial activity of nisin-loaded particles against Gram-negative bacteria *E. coli* and *K. pneumoniae*, as might have been expected, was low (Table 2, Figure 6). Using the agar diffusion assay, semi-transparent lysis zones of 6–7 mm in diameter were detected (Figure 6). Based on MIC analysis, the lowest concentration of nisin–ulvan particles inhibiting the visible growth of *E. coli* bacterium was 50 µg/mL and 100 µg/mL for the *K. pneumoniae*, respectively (Table 2). Our data agree with previous studies revealing that the antimicrobial activity of free or encapsulated nisin is limited to Gram-negative bacteria [39,40].

For the analysis of the efficiency of nisin-loaded particles in real food systems, carrot juice was used. The antimicrobial effect of nisin and nisin-loaded particles against *L. innocua* and *B. subtilis* was evaluated (Figure 7). The viable bacterial count was slightly increased in the control samples with carrot juice when compared to 0.9% NaCl solution. This suggests that carrot juice, due to the high level of nutrients, can support bacterial growth. In addition, the high pH value of carrot juice (from 6.4 to 6.8) makes it challenging to extend the shelf life of this product, as various pathogenic microorganisms are resistant to these pH values [41]. The application of the nisin–ulvan particles into carrot juice resulted in a 4.57 log reduction in *B. subtilis* cells after 24 h incubation of test samples. The observed substantial antimicrobial action of functionalized particles against *B. subtilis* cells in our study was similar in physiological solution and juice. Interestingly, the antibacterial effect of the nisin-loaded particles and free nisin against *L. innocua* cells was significantly enhanced in carrot juice when compared to 0.9 % NaCl solution. Following 24 h incubation of test samples, 4.15 log reduction in *L. innocua* was observed for nisin-loaded ulvan particles, and 4.5 log reduction in *L. innocua* was observed for free nisin; in physiological solution, only about 1 log CFU/mL microbial reduction was recorded. The antimicrobial activity of free ulvan was low but statistically significant against *B. subtilis* cells. However, the ulvan sample had no antimicrobial effect against *L. innocua.* There are several studies on the antimicrobial potential of ulvan with results suggesting it to vary from low to high. Different effects might arise due to the differences in ulvan structure, molecular weight or charge density [23,42]. 

The demonstrated efficient antimicrobial action of nisin-loaded ulvan particles against *L. innocua* and *B. subtilis* in a real food system increases their attractiveness for the preservation of juice against undesirable bacterial contamination. The encapsulated form of nisin is preferable compared to free nisin due to the possibility to ensure the long-term stability of bacteriocin and, as a consequence, the long-term antimicrobial activity. Zimet et al. showed that nisin-loaded nanoparticles produced by alginate–chitosan ionic gelation inhibited *Listeria* *monocytogenes* growth in vacuum-sealed beef samples for twice as long as free nisin [43].

## 4. Conclusions

New nisin-loaded ulvan particles were prepared by the simple and cost-efficient complexation method. The formation of particles was demonstrated by FT-IR spectroscopy and differential scanning calorimetry. The prepared particles exhibited antimicrobial activity, possessing the potential for applications in food preservation. For the first time, the potential of ulvan as a carrier of antimicrobial agent nisin was demonstrated. 

## Figures and Tables

**Table 1:** The efficiency of nisin loading on particles obtained by the complexation ^1^.

The Concentration of Nisin, mg/mL	Encapsulation Efficiency, %			
	pH			
	4.0	5.0	6.0	7.0
0.1	100	100	100	100
0.2	100	100	100	100
0.3	100	100	100	100
0.4	98.5 ± 0.1	100	100	100
0.5	96.2 ± 3.5	100	100	100
0.6	92.6 ± 0.2	86.0 ± 1.3	98.2 ± 0.5	99.9 ± 0.1
1.0	71.5 ± 3.8	75.8 ± 2.4	80.7 ± 4.7	91.8 ± 8.3

^1^ The concentration of ulvan was equal to 0.4 mg/mL.

**Table 2:** Minimum inhibitory concentration of free and encapsulated nisin.

	Minimum Inhibitory Concentration (MIC) of Nisin, µg/mL			
	Gram-Positive Bacteria		Gram-Negative Bacteria	
	*B. subtilis*	*L. innocua*	*E. coli*	*K. pneumoniae*
Free nisin	0.012	0.781	37.5	100
Encapsulated nisin	0.024	1.563	50	100

## Data Availability

The data presented in this study are available on request from the corresponding author.

## Supplementary Materials

The following are available online at https://www.mdpi.com/article/10.3390/foods10051007/s1: Figure S1: Calibration curve for nisin determination. Figure S2: Capillary electropherograms of nisin at different concentrations (0.1–0.4 mg/mL).
