# Supplementary material for: Nisin-Loaded Ulvan Particles: Preparation and Characterization
Source: Foods. 2021 May 4;10(5):1007. doi: 10.3390/foods10051007 (PMC8147952; doi:10.3390/foods10051007)
Supplement: Supplementary file 1 [file foods-10-01007-s001.zip › foods-1192041-supplementary.pdf]

Supplementary Materials

Nisin-loaded ulvan particles: preparation and characterization

Ruta Gruskiene [1], Tatjana Kavleiskaja [2], Ramune Staneviciene [3], Stefanos Kikionis [4], Efstathia Ioannou [4],
Elena Serviene [1,3], Vassilios Roussis [4] and Jolanta Sereikaite [1,*]

[1] Department of Chemistry and Bioengineering, Vilnius Gediminas Technical University, 10221 Vilnius, Lithuania; ruta.gruskiene@vgtu.lt (R.G.); serviene@gmail.com (E.S.)
[2] Institute of Chemistry, Vilnius University, 01513 Vilnius, Lithuania; tania.krivorot81@gmail.com
[3] Laboratory of Genetics, Institute of Botany, Nature Research Centre, 08412 Vilnius, Lithuania; ramune.staneviciene@gamtc.lt
[4] Section of Pharmacognosy and Chemistry of Natural Products, Department of Pharmacy, National and Kapodistrian University of Athens, Panepistimiopolis Zografou, 15771 Athens, Greece; skikionis@pharm.uoa.gr (S.K.); eioannou@pharm.uoa.gr (E.I.); roussis@pharm.uoa.gr (V.R.)
* Correspondence: jolanta.sereikaite@vgtu.lt

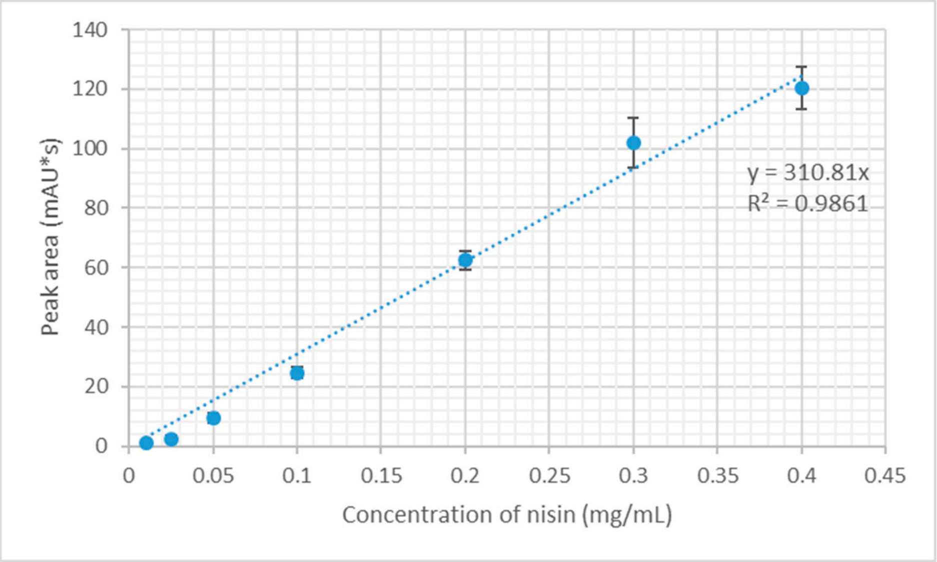

Figure S1. Calibration curve for nisin determination.

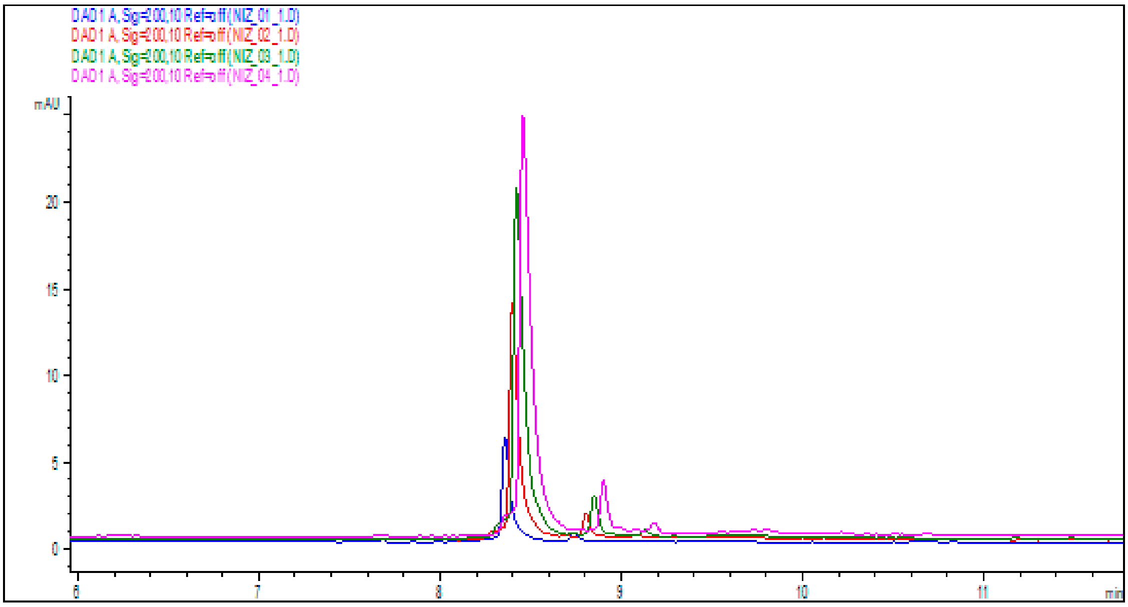

Figure S2. Capillary electropherograms of nisin at different concentrations (0.1-0.4 mg/mL).
